# Medical Items

**Published:** 1911-07

**Authors:** 


### MEDICAL ITEMS

### A GOOD BISMUTH PREPARATION

After an exhaustive study of the chemical and physical properties of bismuth and its compounds, the chemical experts of Parke, Davis & Co., two or three years ago, succeeded in perfecting what many physicians consider the most eligible preparation of the kind—Milk of Bismuth, P. D. & Co., a mixture containing the hydrated oxide of bismuth in suspension. The product is stable under all ordinary conditions of temperature and exposure to light and air.

The advantage which Milk of Bismuth, P. D. & Co., possesses over other compounds of the metal is the state of fine subdivision in which the hydrated oxide is presented. This insures its more thorough distribution over the mucous surface of the alimentary canal, upon which it exerts a peculiarly beneficial effect. Its action is not only astringent but, as some writers have observed, it appears to have a specific effect upon certain lesions, as ulcers, causing them to heal. It is also an antacid and protective, and undoubtedly is mildly antiseptic. Each fluid drachm of Milk of Bismuth, P. D. & C., represents the bismuth equivalent to 5 grains of the sub-nitrate.

### AN IMPROVED HYDRATED MAGNESIA.

An agent which undoubtedly deserves to be more widely employed than it is at present is magnesium oxide. While long held in high professional favor, many physicians in the past have refrained from prescribing it because of the many faulty preparations which found their way upon the market. Practitioners who have felt this restraint would do well to make a test of Milk of Magnesia, P. D. & Co., an improved hydrated magnesia which lacks the objectionable features of many similar preparations and which may be depended upon for uniform and certain results.

Milk of Magnesia, P. D. & Co., is a purely aqueous mixture, concentrated and active, each fluid ounce representing about thirty-two grains of magnesium hydrate. It does not contain sodium sulphate. It is entirely stable under ordinary conditions, remaining unchanged indefinitely. The product is valuable as an antacid and gentle laxative in dyspepsia, sick headache, gout and other complaints attended with hyperacidity and constipation; in diarrhoea due to intestinal fermentation; in gastric disorders peculiar to children in which acidity of the primae viae is often a prominent feature, and whenever gastric irritability and deranged function are present, as evidenced by nausea, gastralgia, eructation, pyrosis and other manifestations of hyperacidity. It is pleasant to take, being readily accepted by children and persons of fastidious taste.

---

### A VALUABLE LOCAL ANAESTHETIC IN ANO-RECTAL SURGERY.

In view of current interest in Quinine and Urea Hydrochloride as a local anaestheic, a report of Dr. Louis J. Hirschman, of Detroit, which appeared in a recent number of the Cincinnati Lancet-Clinic, has peculiar pertinency. Dr. Hirschman reports a total of 102 operations, comprising acute thrombotic hemorrhoids, internal hemorrhoids, interno-external hemorrhoids.

external hemorrhoids, fistula in ano, perineal abscess, fissure in ano, excision of scar tissue, Ball's operation (pruritus ani), hypertrophied papillae, and inflamed Morgagnian crypts. Perfect results were obtained in every case so far as operative anaesthesia was concerned, and in but seven cases was there any post-operative pain. The Doctor uses the one-per-cent. solution in all of his cases of ano-rectal surgery when suturing of the skin is required. The technique of administration is the same as that with weak solutions of cocaine and eucaine.

Dr. Hirschman believes that the substitution of Quinine and Urea Hydrochloride for any of the other anaesthetic salts hitherto employed will prove eminently satisfactory in all cases of ano-rectal surgery in which suturing of the integument is not required. He sums up its advantages as follows: it is soluble in water; it can be sterilized; it is equal to cocaine in anaesthetic power; it is absolutely non-toxic; it has a pronounced hemostatic action; it produces persistent anaesthesia; it is inexpensive.

Quinine and Urea Hydrochloride, in one-per-cent. sterilized solution, is supplied by Parke, Davis & Co., in sealed glass ampoules of five cubic centimeters capacity. An ampoule is opened by breaking off the tip, when the hypodermic needle can be inserted in the neck of the ampoule and the solution drawn into the syringe. Parke, Davis & Co., by the way, issue a sixteen-page brochure on "Local Anaesthesia with Quinine and Urea Hydrochloride," which should be in the hands of every physician and surgeon. The pamphlet details fully the uses of the new anaesthetic, explains the technique of administration, and contains some valuable case reports. A copy may be obtained by writing the company at its home offices in Detroit.

### IF THE STOMACH WERE A SACK.

If the stomach were a cask into which uncooked food and nauseous drugs might be thrown and be digested and absorbed into the system—then there could be no objection to plain crude cod liver oil. The stomach would use it as it would the uncooked food. But since the stomach is not a sack, but happens to be a delicate organ which will resent harsh treatment, uncooked food, nauseous drugs and plain crude cod liver oil are not good for it and against them it rebels. Our common sense warns against uncooked food; deference to the patient's taste guards against the administration of disagreeable drugs, and the manufacturing chemist has made it possible to give cod liver oil in palatable form. Hagee's Cordial of the Extract of Cod Liver Oil Compound is the most efficient and palatable of the cod liver oil preparations and its great value as a tissue food has won for it wide use at the hands of physicians.

---

### THE TREATMENT OF HYDROPHOBIA BY BABIES VACCINE.

Until recently, it has been necessary to send patients exposed to hydrophobia, to a Pasteur Institute (in many instances located in a far distant city) for prophylactic treatment.

The Hygienic Laboratory of the United States Marine Hospital Service devised a method of administering Rabies Vaccine, whereby it could be prepared at a central laboratory, according to Pasteur's method, and distributed to any part of the United States, allowing the patient to be treated by his attending physician.

Briefly, the following is Pasteur's method for preparing Rabies Vaccine: The spinal cord of a rabbit—dead of Rabies as result of an injection of a "fixed virus"—(Rabies Vaccine known to kill within a fixed time) is removed under aseptic conditions. A cord containing the Rabies Virus is suspended over a layer of potassium hydroxide and kept at a temperature of 22 degrees C. from one to eight days. The virus is gradually weakened or attenuated as the cord is dried, the strength being decreased in direct proportion to the extent of the drying.

In the preparation of each injection, a portion of a cord in which the virus has been properly attenuated by drying the requisite number of days, is taken and emulsified by grinding under aseptic conditions with a weak solution of glycerin. The emulsion of Rabies Virus thus prepared constitutes the first dose.

The second dose is prepared in the same manner from a portion of the cord which has not been attenuated to the same degree, and each subsequent dose is prepared in like manner from cords containing virus of increasing potency.

The technique of the administration is quite as simple and safe as the ordinary hypodermatic injection.

H. K. Mulford Company have built and equipped special laboratories at Glenolden, Pa., and, under the personal direction of expert bacteriologists are preparing Rabies Vaccine after the method of Pasteur.

---

### A SAFE AND EFFICIENT ANODYNE

It is not surprising that PASSIFLORA (Daniel's Concentrated Tincture) is rapidly taking the place, in the practices of thousands of physicians, of opium and its derivatives, for it possesses the same soothing qualities of the latter and is free from its danger.

To the man unacquainted with the soporific and anodyne qualities of Passiflora Incarnata (Daniel's Concentrated Tincture), its advantages will prove a revelation. It is free from the dangers of opium and does not enslave the patient in a habit from which escape is well nigh impossible. It does not lock up the secretions, and it may be given over long periods of time with every assurance that it will meet the claims made for it. Not alone is Passiflora efficient substitute for opium, but also for chloral, the bromides and the coal-tar products.

A sample of Daniel's Passiflora may be had by addressing the laboratory of John B. Daniel, Atlanta, Ga.

When dealing with blank cartridge injuries, no consideration of the integrity of the muscles should limit the thorough exposure of every part of the wound; but the larger vessels should be, and the nerves must be, spared.

Eversion of the foot, shortening of the extremity, elevation of the trochanter, spell fracture of the neck of the femur. Manipulation is unnecessary to diagnosis.

In post-operative or other simple retention of urine, even it such devices as enemata, hot water bag over the bladder, the administration of spiritus etheris nitrosi, dipping the hands in hot water, and producng the sound of running water, fail to provoke micturition, catheterization may often be obviated in the female by the simple trick of placing the patient on a well warmed bedpan in which has been poured a little spirits of turpentine.—*American Journal of Surgery.*

